# High Recreational Gamblers Show Increased Stimulatory Effects of an Acute Laboratory Gambling Challenge

**DOI:** 10.1007/s10899-020-09952-3

**Published:** 2020-05-13

**Authors:** L. Miller, A. Söderpalm Gordh

**Affiliations:** 1 Addiction Biology Unit, Department of Psychiatry and Neurochemistry, Institute of Neuroscience and Physiology, The Sahlgrenska Academy, University of Gothenburg, Blå stråket 15, SU/Sahlgrenska, 413 45 Gothenburg, Sweden; 2 Beroendekliniken, Sahlgrenska University Hospital, 413 45 Gothenburg, Sweden

**Keywords:** Recreational gamblers, Subjective effects, Slot machine, Gambling addiction

## Abstract

Gambling in moderation is a socially acceptable behavior and over 60% of the Swedish population gambles every year. It has been seen that slot machines are one of the most addictive and problematic forms of gambling and contribute highly to an addictive behavior. It is unclear why some individuals intensify their gambling behavior over time to extreme levels while others do not. Initial positive response of a drug or as in this case a gambling behavior, most likely influences the likelihood of continuing use in non-addicted individuals. Therefore, we wanted to investigate if recreational gamblers show an altered subjective response to an online gambling challenge, e.g. to casino gambling. The present study was designed to examine the subjective effects after an acute gambling challenge, in healthy recreational gamblers compared with non-gamblers. Eighty-two subjects participated in the study. They were challenged with an acute online slot machine gambling challenge and self-report questionnaires of mood and blood pressure were taken before and after gambling. The gamblers, and more specifically the high recreational gamblers, reported increased stimulative effects after the gambling challenge in comparison to the non-gamblers. Findings suggests that gamblers experience significantly higher arousal effects to an acute online slot machine challenge. This response may be a uniquely predictive behavior for increased risk of gambling addiction.

## Introduction

Gambling addiction is a serious and worldwide problem that affects not only an individual on a personal and familial level but also has an enormous financial consequence (Bergh and Kühlhorn 1994). Around 0.5% of the adult population fulfills the DSM-V criteria for diagnoses (Petry et al. 2005; Kessler et al. 2008) and today the wider concept of problem gambling has been estimated much higher at 0.1–5.8% of the population (Canale et al. 2016). The current literature also demonstrates that the prevalence of problem gambling in areas of high gambling incidence is extraordinarily high. With figures as high as 5% and much higher within certain group’s for instance young adults and people who are suffering with mental health disorders (Shaffer et al. 1999).

One of the biggest changes to gambling behavior over the past decade has been the widespread increase of online casinos and the ever-increasing availability of internet gambling (Bonnaire 2012). It has been seen that online slot machines are one of the most addictive and problematic forms of gambling and contribute highly to casino turnover (Mcbride and Derevensky 2012; Dixon et al. 2018). Access to internet gambling also allows people to engage in gambling at any time and place and is allowing more and more people to easily become addicted (Currie et al. 2013; Gainsbury et al. 2015; Chóliz 2016).

It is unclear why some individuals intensify their gambling behavior over time to extreme levels while others do not. Initial positive response of a drug, or as in this case a gambling behavior, most likely influence the likelihood of continuing use in non-addicted individuals (Haertzen et al. 1983; De Wit and Phillips 2012). One of the initial responses is the subjective effects associated with gambling. Previous literature on gamblers describes the phenomenon of increased arousal in situations associated with gambling (Carroll and Huxley 1994; Sharpe et al. 1995; Blanchard et al. 2000; Meyer et al. 2000). This is also interesting in regards to the literature that is well known to the field of subjective effects of alcohol.

In the line of alcohol research, it is well established that individuals show differences in the subjective responses to alcohol and there are several theories suggesting that a specific subjective drug response play an important role in the developmental and maintenance of addiction. An earlier theory proposed that a low level of response, such as a lesser subjective behavioral responses to alcohol motivates one to drink more, predicts a risk for developing an alcohol use disorder (Schuckit 1994). More recently it has been proposed that experience of positive and stimulant-like effects of alcohol, is associated with greater ratings of drug liking and euphoria motivating the subject to drink more (de Wit et al. 1987; Chutuape and de Wit 1994). The theory about a greater response to the drug, more specifically the rewarding and stimulating effects, has been found to be better and a more intuitive predictor of the risk of developing an alcohol use disorder (Wise and Bozarth 1987); Newlin et al. 2010; King et al. 2019). Further, a higher rather than a lower response to alcohol predicted a development of an alcohol use disorder in a 5-year prospective study. This effect was specifically pronounced in heavy drinkers (King et al. 2014, 2019).

Studies on gamblers have previously seen subjective arousal but mainly found autonomic stimulation in response to a gambling challenge. Both pathological and regular gamblers have shown increased subjective arousal compared to non-regular gamblers playing on slot machines (Anderson and Brown 1984; Griffiths 1995; Brown et al. 2004; Sharpe 2004). Furthermore, problem gamblers exposed to real life casino gambling has also been seen to show increased heart rate and norepinephrine levels compared to non-problem gamblers in both men and women (Meyer et al. 2004; Yuchaet al. 2007). In a laboratory pilot study, male problem gamblers had been found to show increased heart rate in comparison to controls while listening to individualized tapes of the gamblers' preferred form of gambling (Blanchard et al. 2000). On the contrary, young dependent slot machine players did not differ in heart rate activity after exposure to real life gambling compared to nondependent gamblers (Carroll and Huxley 1994). Also in the laboratory and in line with Carroll and Huxley’s study, when problem gamblers were asked to recall a gambling winning situation they did not show an increase in heart rate that differed from regular or casual gamblers. They did however display a greater reactivity in muscular activity and skin conductance (Sharpe et al. 1995).

Previous research has further studied the neurochemical mechanisms underlying reward and reinforcement in pathological gamblers. Dopamine has been seen to play an important role just as in alcohol addiction. In the cerebrospinal fluid (CSF) obtained from pathological gamblers, Bergh et al. (1997) found a decrease in dopamine and an increase in the dopamine metabolites DOPAC and HVA, compared to controls, suggesting a role for increased dopamine turnover in this disorder. Meyer et al. (2004) also found acute elevated dopamine levels in response to casino gambling in problem gamblers. Previous research also shows that gambling can induce amphetamine (AMPH) like effects (Zack and Poulos 2004). found that AMPH primed a motivation to gamble and that the severity of problem gambling predicted the AMPH-induced motivation. On the other hand, Boileau et al. (2013) found no difference between pathological gamblers and healthy controls in D2/D3 levels measured in a positron emission tomography. Furthermore, in rats, chronic exposure to a gambling-like schedule of reward by a sucrose solution promoted amphetamine sensitization much like an exposure to amphetamine itself (Zack et al. 2014). These studies propose a dopamine reward sensitivity in gamblers similar to the ones described in substance use disorders.

The evidence that gamblers show elevated subjective arousal, a stimulation of cardiovascular activity (for review see Goudriaan et al. 2004) and elevated dopamine levels in response to gambling supports the theory of a greater stimulative drug-like effect in gamblers. Acute gambling challenge studies may help to identify risk factors predicting future use. Initial positive or negative effects of gambling probably influence the likelihood of continued use at least in the short term.

In both human and animal drug research there is also evidence of high responders and low responders. Animals who are high responders show predictive behaviours to a variety of drugs such as d-amphetamine, cocaine, morphine, alcohol and nicotine (for review see Kabbaj 2006). In humans, high responders to a stress task (i.e. high cortisol responders) show for example increased vulnerability to infectious diseases (Kirschbaum et al. 1995), they consume more food (Epel et al. 2001), they are higher DA responders and experience positive effects of amphetamines to a higher degree than low cortisol/DA responders do (Wand et al. 2007). They also show a dose dependent increased response to an acute administration of alcohol (Brkic et al. 2016). In gamblers, a high frequency gambler (> 3 times/week) has been found to show cue induced increases in heart rate and arousal before gambling in comparison to a low frequency gambler (< 1time/month, Leary and Dickerson 1985). Further, frequent gamblers (> 3 times/week) have been found to show increased heart rate after playing on a slot machine compared to infrequent gamblers (1–2 times/month) and non-gamblers (Moodie and Finnigan 2005). In our study we chose average time (minutes) spent gambling per week. This was based on studies having length of time spent gambling as an indicator of problematic gambling (Currie et al. 2008; Canale et al. 2016; Joyce et al. 2019). Longer periods of time, dollars spent each day and frequency are all indicators of an at risk gambling behavior (Currie et al. 2013). A high frequency gambler (> 6 time/week) has further experimentally been found to show increased resistance to a partial reinforcement schedule compared to a low frequency gambler (< 1 time/week, Horsley et al. 2012). Whether high recreational gamblers, differed by time, show an altered subjective response to an online gambling challenge in comparison to controls has to our knowledge not been investigated.

In order to determine subjective effects in underlying gambling addiction we aim to acutely challenge recreational gamblers and non-gambling individuals to an online casino task where you can “win” a fake monetary reward. Further, building on evidence that gamblers show elevated arousal both subjectively and cardiovascular (Griffiths 1995; Brown et al. 2004; Yucha et al. 2007), and in addition show altered dopamine activation in response to gambling (Bergh et al. 1997; Meyer et al. 2004), we suggest that “high” recreational gamblers show greater effects to a gambling challenge than “low” recreational gamblers do in comparison to controls.

Therefore, our first hypothesis was that recreational gamblers showed increased subjective and cardiovascular effects after a computerized gambling casino challenge in comparison to non-gamblers. Based on previous research (Leary and Dickerson 1985; Moodie and Finnigan 2005; Wand et al. 2007), our second hypothesis was that high recreational gamblers show stronger increased subjective and cardiovascular effects after a computerized gambling casino challenge in comparison to low recreational gamblers and controls.

## Method

### Subject Recruitment and Screening

Eighty-two healthy recreational gamblers and non-gamblers (n = 35 men, n = 47 women) recruited via advertisements were initially screened by telephone for major eligibility criteria. Participants were invited to the laboratory for further screening upon meeting the eligibility requirements of: age (19–65), normal BMI (18.5–25), moderate consuming of alcohol, (i.e. no more than 9 standard drinks per week for women and 12–14 for men), negative history of substance abuse and or negative history of somatic diseases. Subjects completed the DSM-V diagnoses for gambling addiction (American Psychiatric Association: Diagnostic and Statistical Manual of Mental Disorders) and did not currently receive treatment for gambling addiction. They also answered questions about time in minutes they spent gambling each week, and on average, how much money they would have spent on a regular gambling day in the last 3 month. Alcohol Use Disorders Identification Test (Saunders et al. 1993; Babor et al. 1992) and the psychiatric symptom checklist **(**SCL-90; Derogatis 1994) assessing medical and psychiatric histories. The study was approved by the regional ethics committee of the University of Gothenburg and complied with the guidelines of the Declaration of Helsinki.

### Design and Procedure

The study was conducted in a comfortable environment, furnished like an apartment living room (see section laboratory setting). Subjects were always run alone. The session procedure was as follows: Subjects arrived at the laboratory between 09:00 and 16.00 h. Objective measures of blood pressure (BP) and subjective self-report measures of The Drug Effects Questionnaire (DEQ) and the Addiction Research Inventory Scale (ARCI) were assessed at baseline (0 min). Participants were then asked to virtually play on an online casino gambling program on a computer for 10 min until an alarm sounded which indicated the end of the gambling period. The starting point was a credit of SEK 75 (1 credit = SEK 1), and every spin on the slot-machine simulation let the participant gamble for 1, 2 or 3 credits. The participants were unaware of the length of time for which they gambled. Directly after playing on the slot machine the last measures of the subjective and the objective measures were taken again. At the end of the study, participants were debriefed by the experimenter and received compensation for their participation.

#### Gambling Model

Each participant was presented with the online casino All JSlots, version 2.2. on a laptop. This slot machine mimics the exact version that is available online and is complete with all reel features. The specific feature with the slot machine is that wins are positively reinforced via classical conditioning, which facilitates the development of problem gambling. Jackpot wins and losses, flashing sequences and music were accompanied with line wins, along with brightly coloured imagines of a mix of cherry’s, oranges, motorcycles and various other imagines. The screen displays the ‘payline’, total credits, bet and amount that the winner can be paid out. This simulates online slot machines, as participants were able to decide for themselves if they would like to bet one, two or three credits, which represented the amount of lines played. Participants played on average 55 spins each time. The game was randomised and each person achieved a different amount of ‘wins or losses’ similar to a real online slot machine. Subjects played only for slot machine credits, which were not paid out in cash.

## Laboratory Environment

The study was conducted in a laboratory setting at Sahlgrenska University hospital in Gothenburg Sweden as part of the Addiction Biology Unit (ABU), Section for Psychiatry and Neurochemistry, Institute of Neuroscience and Physiology. The laboratory setting was designed to look and feel like a living room setting to simulate how people would online gamble at home. The room comprised of a large sofa, table, armchairs, curtains, bookshelf, paintings on the wall, kettle and magazines. Participants were asked not to work, study or use their phones. When they had finished their paperwork, they were told to relax and wait for the next step in the process of the study.

### Dependent Measures

Dependent measures included physiological effects as described below. The primary dependent measure was the Drug Effect Questionnaire (DEQ). Physiological measures were heart rate and blood pressure.

### Self-reported and Objective Measures

Participants were screened using a variety of standardized questionnaires primarily used for drug abuse. All questionnaires were used in a version translated to Swedish. The questionnaires used to assess mood states and subjective drug effects described below are sensitive to the effects of a variety of psychoactive drugs (e.g. de Wit and Griffiths 1991; Fischman and Foltin 1991).

*The Drug Effect Questionnaire (DEQ)* assesses the extent to which subjects experience drug effects (Johanson and Uhlenhuth 1980; Fischman and Foltin 1991; Morean et al. 2013). The DEQ is widely used as a screening method for drug abuse and risk of abuse. It allows the participant to evaluate the effect of a specific drug using four sub-categories: effect—DEQ (do you feel an effect?; None at all to A lot), like—DEQ (do you like the effect?; Not at all to Like very much), high—DEQ (do you feel a high?; Not at all to Very much), and want—DEQ (do you want more?; Not at all to Very much). In every category, the individuals rated on a 100 mm labeled magnitude scale. The DEQ was used post-testing only.

The *Addiction Center Research Inventory* (ARCI) (Haertzen 1966; Hickey et al. 1986; Martin et al. 1971). The ARCI is a 49-item true–false questionnaire designed to assess subjective responses to different categories of abused drugs. It consists of five subscales: sedation (pentobarbital-chlorpromazine group (PCAG)), stimulant-like effects (amphetamine (A) and Benzedrine group (BG)), somatic and dysphoric effects (lysergic acid (LSD)), and euphoria (Morphine-Benzedrine group (MBG)).

ARCI was used to measure subjective responses to gambling. We focused our analyses on the Benzedrine Group, euphoric effects and Amphetamine-like, stimulant effects scales, as these represent the typical positive, rewarding effects of amphetamine (for reviews summarizing evidence that these scales are sensitive to the acute effects of amphetamine and that they are predictive of amphetamine choice, (see Fischman and Foltin 1991; Jasinski 1991; de Wit and Phillips 2012). The ARCI has also been proven sensitive to the effects of gambling. In a study with nineteen volunteers with histories of compulsive gambling they found that “simulated winning at gambling” produced a euphoria similar to the euphoria induced by the psychoactive drugs of abuse, particularly psychomotor stimulants (Hickey et al. 1986).

### Physiological Measures

*Heart rate and blood pressure* A *Dynamap®* monitor was used to monitor heart rate and blood pressure. Measurements were taken prior to and directly after slot-machine gambling.

Primary outcome measures were the feel, like, high, and want more scales from the DEQ. In addition, we focused on the stimulant (A scale) and euphoria (MBG) subscales of the ARCI. These scales represent the typical rewarding and hedonic effects of drugs.

### Statistical Analyses

For descriptive purpose mean and standard error of mean (SEM) were presented for continuous variables. The one way ANOVAs and descriptive analysis was performed using IBM Statistical Package for Social Sciences software version 25.0 (SPSS, Inc. 2017). The other tests were performed using SAS 9.4 (SAS Institute, Inc., Cary, NC). Data was analyzed using mixed models, response profile analysis. The significance level was set at the 0.05 and corrections for multiple comparisons were made with a Bonferroni test. For the first hypothesis, a split Currie et al. (2008) was made between Recreational Gamblers (RG; > 15 min/week) and Non-Gamblers (Non-G, 0 min/week). A one-way ANOVA was performed to test baseline differences between the two groups. The following analysis was performed with General Linear Model (GLM), test of between subject effects. The analyses were built on two groups (RG and Non-G), and the difference between the two time points baseline (0 min) and post test (+ 10 min) was used in to test the differences on the DEQ (Effect, High, Like and Want more), the ARCI-scale (PCAG, A, BG, LSD, MBG) and blood pressure (systolic, diastolic and pulse). For the second hypothesis, a new split Currie et al. (2008) was then made between High Recreational Gamblers (HRG; gambles for money > 60 min a week), Low Recreational Gamblers (LRG; < 60 min/week) and Non-gamblers (Non-G; 0 min/week). The analyses were therefore built on three groups (HRG and LRG or Non-G), and the difference between the two time points baseline (0 min) and post test (+ 10 min). A General Linear Model (GLM), test of between subject effects was used to test gamblers (HRG, LRG, Non-G) on the variables of DEQ (Effect, High, Like and Want), the ARCI-scale (PCAG, A, BG, LSD, MBG) and blood pressure (systolic, diastolic and pulse). Adjusted analyses were obtained by using logistic regression with grouping variable as dependent variable, post-baseline value or difference from baseline to post-baseline value as main effect variable and baseline value as covariate for both hypothesis one and two.

## Results

### Subject Demographics

The demographic characteristics, drug use and gambling data between Recreational Gamblers (RG) and Non-Gamblers (Non-G), High Recreational Gamblers (HRG), Low Recreational Gamblers (LRG) and Non-Gamblers (Non-G) are shown in Table 1. The mean age in the RG group was 27.7 and in the Non-G it was 24.6. The mean weight in the RG group was 72.1 kg and in the Non-G it was 68.2 kg. All of the subjects were Caucasian and the subjects belonging to the different groups did not significantly differ on any of the demographic or drug use variables obtained. Further, no significant demographic differences were found between the HRG’s and the LRG’s.

**Table 1:** Demographics and drug use data between recreational gamblers (RG), non-gamblers (Non-G), high recreational gamblers (HRG), low recreational gamblers (LRG)

	Gamblers (n = 47)	Non-gamblers (n = 35)	HRG (n = 22)	LRG (n = 24)	Non-Gamblers (n = 35)
Female (n)	27	20	9	18	20
Male (n)	20	15	14	6	15
Age (years, mean ± SEM)	27.7 ± 1.2	24.6 ± 1.0	27.2 ± 1.4	28.1 ± 0.0	24.6 ± 1.0
Weight (kg, mean ± SEM)	72.1 ± 1.2	68.2 ± 2.3	75.1 ± 2.5	69.3 ± 2.1	68.2 ± 2.3
*Race/ethnicity*					
Caucasian	44	33	21	22	34
Asian	1	1	0	0	0
Hispanic Other	1 1	0 1	0 2	0 2	0 1
*Education (n)*					
High School grad or less	2	1	0	2	1
College student	28	25	10	16	25
College graduate	16	9	8	10	9
*Current gambling situation*					
DSM-IV (total points, mean ± SEM)	0.4 ± 0.1	0.02 ± 0.0	0.4 ± 0.2	0.36 ± 0.2	0.02 ± 0.0
Gambling (minutes/week, mean ± SEM)	134 ± 7	0.0 ± 0.0	197 ± 8	17 ± 3	0.0 ± 0.0
“Bets” (dollars/day)	6.4 ± 2.7	0.0 ± 0.0	10.4 ± 6.0	2.57 ± 1.0	0.0 ± 0.0
Computer gaming (yes)	17	7	10	7	7
*Current drug use*					
AUDIT (total points, mean ± SEM)	5.3 ± 0.5	5.4 ± 0.5	5.9 ± 0.7	4.8 ± 0.7	5.4 ± 0.5
Alcohol drinks (n/week, mean ± SEM)	3.9 ± 0.7	3.0 ± 0.5	5.3 ± 1.2	2.7 ± 0.6	3.0 ± 0.5
Caffeine (n users)	31	24	17	13	24
Cups of coffee (n/day)	10.7 ± 1.5	8.1 ± 1.6	11.2 ± 2.0	10.2 ± 2.3	8.1 ± 1.6
Cigarette consumers on a daily basis (n users)	4	2	2	2	2
Cigarettes consumed (n/day)	1.7 ± 1.3	0.2 ± 0.1	3.3 ± 2.8	0.2 ± 0.2	0.2 ± 0.1
Snuff consumers on a daily basis (n users)	6	4	4	2	4
Snuffs consumed (n/day)	8.1 ± 4.1	7.7 ± 4.3	8.7 ± 4.4	1.1 ± 1.1	7.7 ± 4.3

The demographics are presented as arithmetic mean ± standard error of mean (SEM)

### Subjective and objective effects of gambling between recreational gamblers and non-gamblers

First, a one-way between subjects ANOVA was conducted to compare the pre conditions between the RG’s (n = 35) and Non-G’s (n = 47) before the gambling challenge on the main dependent variables, systolic blood pressure F(1,80) = 0.002, *p* = ns; diastolic blood pressure F(1,80) = 1.50, *p* = ns; puls F(1,80) = 0.22, *p* = ns; ARCI-AMPH F(1,80) = 0.91, *p* = ns; ARCI-MORPH F(1,80) = 2.24, *p* = ns; ARCI-LSD F(1,80) = 0.48, *p* = ns; ARCI-BENZ F(1,80) = 1.28, *p* = ns; ARCI-PENT F(1,80) = 0.02, *p* = ns; DEQ “Effect”, “High”, “Like” and “Want more” no data shown since baseline was set to zero. No statistical significant differences were found between any of the measures. It is important to note that the two groups did not differ at baseline at any of the main measures. Table 2 shows means ± SEM and the differences for the dependent measures and groups.

**Table 2:** Arithmetic mean ± standard error of mean (SEM) and differences score for all dependent measures taken in the study

Dependent measures		Gamblers (n = 47)			Non-gamblers (n = 35)		
*DEQ*	Pre	Post	Diff	Pre	Post	Diff	Pre
Effect	0.0 ± 0.0	35.5 ± 4.0	35.5 ± 4.0 *	0.0 ± 0.0	24.1 ± 3.2	24.1 ± 3.2	0.0 ± 0.0
High	0.0 ± 0.0	24.5 ± 3.6	24.5 ± 3.6 *	0.0 ± 0.0	12.4 ± 3.1	12.4 ± 3.1	0.0 ± 0.0
Like	0.0 ± 0.0	41.9 ± 4.0	41.9 ± 4.0	0.0 ± 0.0	32.1 ± 4.6	32.1 ± 4.6	0.0 ± 0.0
Want more	0.0 ± 0.0	24.1 ± 3.8	24.1 ± 3.8	0.0 ± 0.0	17.1 ± 3.1	17.1 ± 3.1	0.0 ± 0.0
*ARCI*							
Amphetamine	3.8 ± 0.3	3.7 ± 0.3	3.8. ± 0.1	3.4 ± 0.3	3.7 ± 0.4	-0.3 ± .4.0	4.3 ± 0.4
Morphine	5.7 ± 0.4	5.5 ± 0.4	0.2 ± 4.0	4.6 ± 0.5	4.8 ± 0.5	-0.1 ± 5.0	5.6 ± 0.6
LSD	3.4 ± 0.2	3.1 ± 0.2	0.2 ± 2.0	3.1 ± 0.2	3.1 ± 0.3	-0.0 ± .3.0	3.5 ± 0.4
Benzedrine	3.9 ± 0.2	3.6 ± 0.2	0.2 ± 0.1 *	3.4 ± 0.3	4.1 ± 0.3	-0.7 ± 0.2	3.9 ± 0.4
*BP*							
Systolic	121.4 ± 1.7	118.0 ± 0.2	5.8 ± 3.3	121.3 ± 2.8	116.6 ± 2.3	4.6 ± 1.6	124.1 ± 2.0
Diastolic	79.3 ± 1.3	76.2 ± 1.4	4.6 ± 1.9	76.7 ± 1.6	74.5 ± 1.3	-2.2 ± 1.4	79.5 ± 1.9
Pulse	71.4 ± 1.2	67.8 ± 1.2	5.0 ± 1.7	72.4 ± 1.9	70.7 ± 1.6	3.8 ± 2.3	71.6 ± 1.5

Dependent measures	HRG (n = 23)			LRG (n = 24)			Non-gamblers (n = 35)	
*DEQ*	Post	Diff	Pre	Post	Diff	Pre	Post	Diff
Effect	43.1 ± 5.4	43.1 ± 5.4 *	0.0 ± 0.0	28.5 ± 5.5	28.5 ± 5.0 *	0.0 ± 0.0	24.1 ± 3.2	24.1 ± 3.2
High	29.0 ± 5.5	29.0 ± 5.5 *	0.0 ± 0.0	20.3 ± 4.6	20.3 ± 4.6	0.0 ± 0.0	12.4 ± 3.1	12.4 ± 3.1
Like	43.3 ± 6.4	43.3 ± 6.4	0.0 ± 0.0	46.6 ± 5.8	40.6 ± 5.8	0.0 ± 0.0	32.1 ± 4.6	32.1 ± 4.6
Want more	28.7 ± 6.4	28.7 ± 6.6	0.0 ± 0.0	19.8 ± 4.2	19.8 ± 4.2	0.0 ± 0.0	17.1 ± 3.2	17.1 ± 3.2
*ARCI*								
Amphetamine	4.0 ± 0.4	0.2 ± 0.4	3.4 ± 0.4	3.3 ± 0.4	0.0 ± 0.4	3.4 ± 0.3	3.7 ± 0.4	-0.3 ± 0.4
Morphine	5.8 ± 0.7	-0.1 ± 0.7	5.7 ± 0.7	5.2 ± 0.6	0.5 ± 0.5	4.6 ± 0.5	4.8 ± 0.5	-0.1 ± 0.5
LSD	3.1 ± 0.3	0.3 ± 0.3	3.2 ± 0.3	3.0 ± 0.4	-0.7 ± 0.2	3.1 ± 0.2	3.1 ± 0.3	-0.0 ± 0.3
Benzedrine	3.9 ± 0.3	0.4 ± 0.2 *	3.8 ± 0.0	3.8 ± 0.3	0.0 ± 0.3 *	3.4 ± 0.3	4.1 ± 0.3	-0.7 ± 0.2
*BP*								
Systolic	118.1 ± 2.2	10.5 ± 6.0	118.8 ± 2.6	117.4 ± 3.3	1.4 ± 3.1	121.3 ± 2.8	116.6 ± 2.3	4.6 ± 1.6
Diastolic	75.4 ± 1.8	7.3 ± 3.6	79.1 ± 1.8	117.0 ± 3.3	2.1 ± 1.4	76.7 ± 1.6	74.5 ± 1.3	2.2 ± 1.4
Pulse	66.9 ± 1.4	7.6 ± 3.0	71.1 ± 71.8	68.6 ± 1.9	2.5 ± 1.5	72.4 ± 1.9	70.7 ± 1.6	3.8 ± 2.3

Data are presented as pre, post test and difference score. Significant differences are denoted with an asterisk indicating differences in comparison with the Non-gamblers (Non-G)

**Fig. 1:**
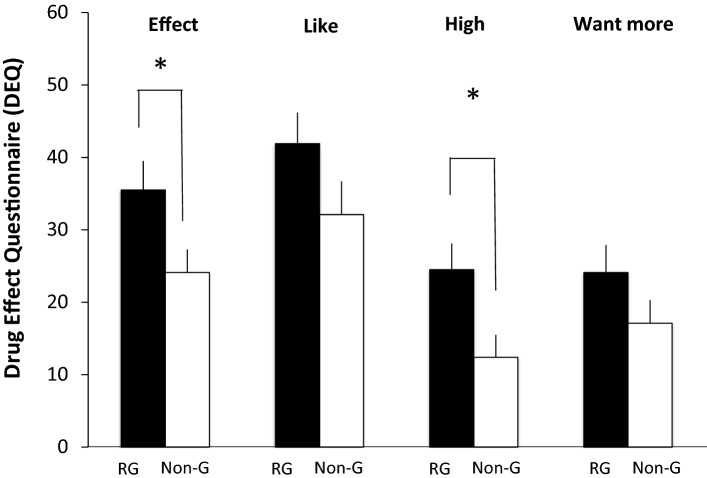
Mean difference scores ± standard error of mean (SEM) on the Drug Effects Questionnaire (DEQ) between pre and post test for Effect, High, Like and Want more after the 10 min electronic slot machine challenge between the Recreational gamblers (RG; black bars) and the Non gamblers (Non-G, white bars). The asterisks denote significant differences between pre and post test (*p* < 0.05)

**Fig. 2:**
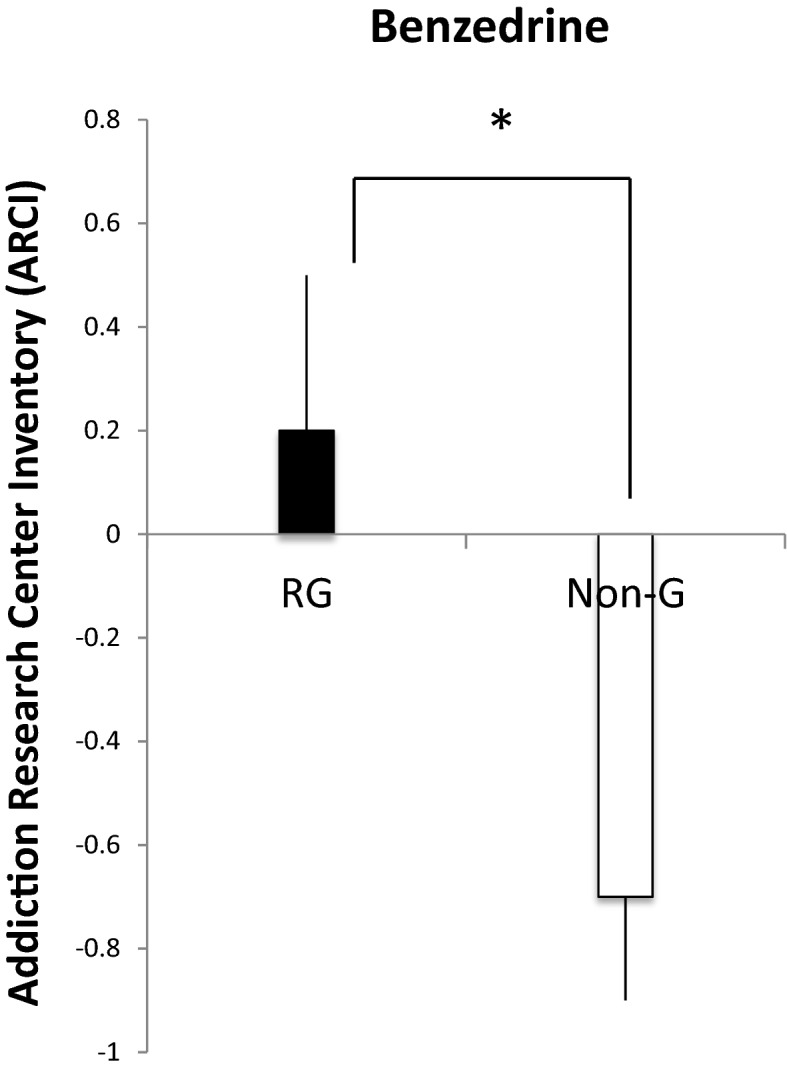
Mean difference scores ± standard error of mean (SEM) on the Addiction Research Center Inventory (ARCI) between pre and post test for Benzedrine scale after the 10 min electronic slot machine challenge between the Recreational gamblers (RG; black bars) and the Non gamblers (Non-G, white bars). The asterisk denote significant differences between pre and post test (*p* < 0.05)

In the first hypothesis, it was stated that RG’s would show increased subjective effects after a gambling slot machine challenge in comparison to Non-G’s. In a General Linear Model (GLM), a test of between subject effects, we found that on the DEQ measure “Effect” a significant enhancement was found between RG’s and Non-G’s F(1,77) = 4.54, *p* = 0.04. A significant difference between RG’s and Non-G’s was further found with the same analysis on DEQ “High” F(1,77) = 5.72, *p* = 0.02. RG’s reported feeling increased “Effect” and “High” after the gambling challenge in comparison to the Non-G’s (Fig. 1). No significant differences were found on “liking” and “want more” although visual trend shows that RGs want to play more than Non-G’s do. A significant effect on ARCI-Benzedrine scale was also found between RG’s and Non-G’s F(1,82) = 7.53, *p* = 0.007 indicating that RG’s found the gambling to be Benzedrine-like (i.e. amphetamine like; Fig. 2). No significant differences were found on either of the blood pressure measures.

### Subjective and objective effects of gambling between high recreational gamblers, low recreational gamblers and non-gamblers

First, a one-way between subjects ANOVA was conducted to compare the pre conditions between the HRG’s (n = 23), LRG’s (n = 24) and Non-G’s (n = 35) before the gambling challenge on any of the main dependent variables, systolic blood pressure F(2,79) = 0.81, *p* = ns; diastolic blood pressure F(2,79) = 0.75, *p* = ns; pulse F(2,79) = 0.12, *p* = ns; ARCI-AMPH F(2,79) = 1.50, *p* = ns; ARCI-MORPH F(2,79) = 1.11, *p* = ns; ARCI-LSD F(2,79) = 0.35, *p* = ns; ARCI-BENZ F(2,79) = 0.64, *p* = ns; ARCI-PENT F(2,79) = 0.03, *p* = ns; DEQ no data shown since baseline was set to zero. The three groups did not differ at baseline at any of the main measures. Table 2 shows means ± SEM and the differences for the dependent measures and groups.

**Fig. 3:**
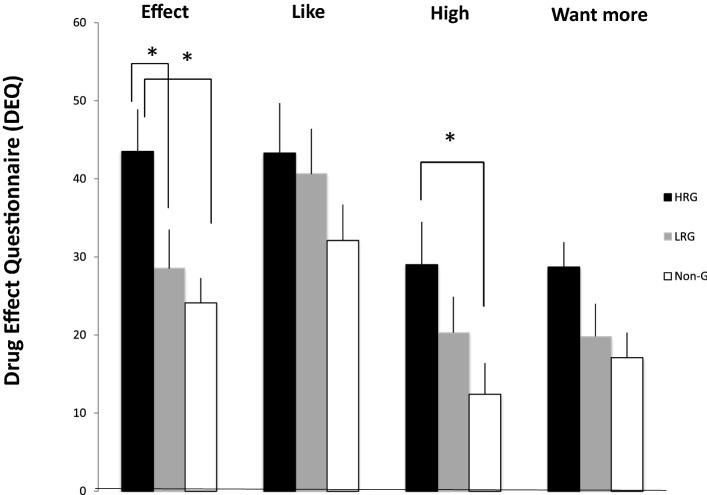
Mean difference scores ± standard error of mean (SEM) on the Drug Effects Questionnaire (DEQ) between pre and post test for Effect, High, Like and Want more after the 10 min electronic slot machine challenge between the High Recreational gamblers (HRG; black bars), the Low Recreational gamblers (LRG, grey bars) and the Non gamblers (Non-G, white bars). The asterisks denote significant differences between pre and post test (*p* < 0.05)

**Fig. 4:**
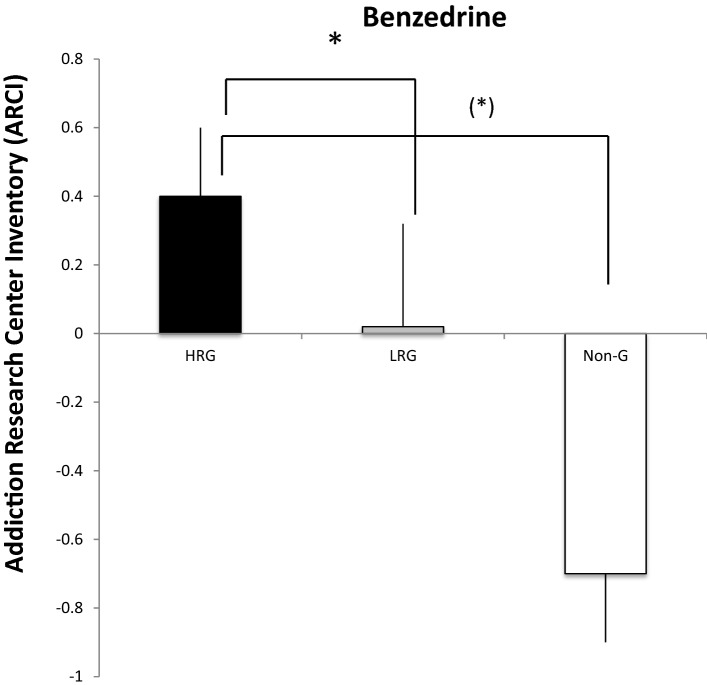
Mean difference scores ± standard error of mean (SEM) on the Addiction Research Center Inventory (ARCI) between pre and post test for Benzedrine scale after the 10 min electronic slot machine challenge between the Recreational gamblers (RG; black bars), the Low Recreational gamblers (LRG, grey bars) and the Non gamblers (Non-G, white bars). The asterisk denote significant differences between pre and post test (*p* < 0.05)

Our second hypotheses stated that HRG’s showed more intense subjective dopamine like effects such as “high” after the gambling challenge. In a General Linear Model (GLM) procedure we found that on the DEQ measure “Effect” a main significant was found F(3,80) = 3, *p* = 0.03. In a further second order analysis between the three groups it was found that HRG’s differed from Non-G’s (*p* = 0.004), and that the LRG’s also differed from the Non-G’s (*p* = 0.04). No significant difference was found between HRG’s and LRG’s. In a further GLM analysis a main effect of the DEQ “High” was found F(3,80) = 2.82. *p* = 0.04. It was found that the HRG’s significantly differed from Non-G’s (*p* = 0.005). No other group comparison differed significantly from each other (Fig. 3). On the DEQ “Liking” and “Want more” no main effects was found and therefore no further analysis was made between the three groups. In a General Linear Model (GLM) procedure a significant main effect on ARCI-Benzedrine scale was found F(3, 81) = 2.74, *p* = 0.04. A second order analysis found that the LRG’s significantly reported a higher score than the HRG’s (*p* = 0.009; Fig. 4) but none of the groups differed significantly from the Non-G’s. However, a second order analysis revealed a trend towards a difference between HRG’s and Non-G’s *p* = 0.06. No other group comparison differed significantly from each other. No significant differences were found on either of the blood pressure measures between the three groups.

## Discussion

The present study describes subjective and cardiovascular effects after an acute gambling challenge in recreational gamblers and non-gamblers. First, it was found that the Recreational Gamblers showed increased subjective “effect” and “high” in comparison to the Non-Gamblers. Recreational Gamblers also showed an increased Benzedrine-like effect. Secondly, when we analyzed both the High and Low Recreational Gamblers it was found that the High Recreational Gamblers boosted the drug-like response (effect and high) in comparison to the Non-Gamblers. We did not see any cardiovascular arousal between either groups.

Factors contributing to a gambling reinforcement behavior are important for understanding the risk of developing a gambling problem. Pathological gambling shares many similarities with alcohol addiction in terms of clinical phenomena (e.g. craving, tolerance, compulsive use, loss of impulse control) and heritability (Slutske et al. 2010; Blanco et al. 2012). Pathological gamblers also show a similar arousal and reward sensitivity during gambling probably mediated by the mesolimbic dopamine system well known to the field of alcohol addiction (Yau and Potenza 2015).

The rewarding properties of the acute gambling challenge in our study seemed to be strong enough that even a recreational gambler who “only” gambles for money on average 2 h a week significantly feels a drug-like effect while gambling during 10 min compared to a non-gambler. Regular gamblers showed increased drug-like “effect” and “high” and visual increases on “liking” and “want more”. Regular gamblers further showed an increase on the Benzedrine scale on the ARCI in comparison with Non-Gamblers. The subscale is used as a proxy for stimulant-like effects produced by amphetamines and the scale has been widely used to assess the abuse potential of drugs (Martin et al. 1971; Haertzen et al. 1983). Our findings are also in line with previous research describing problem gambler’s subjective arousal compared with non-regular gamblers in challenge studies (Anderson and Brown 1984; Griffiths 1995; Brown et al. 2004; Sharpe 2004). As previously postulated for gamblers, they have also been found to report greater preference for amphetamines and a greater desire to gamble after a priming dose of amphetamine (Zack and Poulos 2004). Alteration in dopamine in problem gamblers (Meyer et al. 2004) may reflect the stimulative drug-like effects seen in our study. These studies provide evidence for shared important mechanisms between stimulant drug-like effects of gambling and the rewarding properties of a psychostimulant. Our findings could also be compared to a social drinker that is challenged with a high dose of alcohol. King et al. has in a series of experiment showed that high social drinkers exhibit greater stimulant and rewarding responses to a single alcohol challenge compared to low drinkers (King et al. 2011). They also showed that the sensitivity to the stimulant effects of alcohol predicts future alcohol problems both at two years (King et al. 2011) and 6 years after the initial alcohol challenge (King et al. 2014). However, to which extent the combined stimulative drug-like subjective effects seen in our study are associated with maintenance of a gambling behavior or its relation to an abuse potential (de Wit et al. 1987; Chutuape and de Wit 1994) needs to be further studied.

In the present study, our Regular Gamblers did not show greater cardiovascular reactivity compared to Non-Gamblers. Previous research has found cardiovascular activity to be an unreliable measure of arousal and reward in gamblers. Both laboratory studies and real life situations have shown inconclusive results on cardiovascular activity. Gamblers have previously been found to show moderate increases in heart rate as a result of casino gambling (Anderson and Brown 1984; Leary and Dickerson 1985; Blanchard et al. 2000; Meyer et al. 2000). However, our current result is in line with observations that did not find increased arousal in heart rate (Carroll and Huxley 1994; Sharpe et al 1995). Confounds such as sensation seeking and loss of control, originally studied by Zuckerman (1984) and Zuckerman and Neeb (1979), baseline resting and movement during gambling (Meyer et al. 2000), habituation to the experimental condition (Eifert and Heffner 2003) all have an effect of heart rate and needs to be taken in consideration in further studies. Gamblers in our study may have had an elevated baseline heart rate, our choice of online slot machine could play a role in that the gambling session was not stimulating enough, or that the short period of the gambling time had no effect on cardiovascular activity. The ecological validly in our study can also be challenged. Playing for real money in a real life casino environment may have enhanced our cardiovascular results. A future need for more stringent indicators and the role cardiovascular arousal plays in maintaining a gambling behavior needs to be studied further.

In our second hypothesis we found that High Recreational Gamblers (those who gambled almost three hours a week) were the ones that mainly boosted the stimulative drug-like properties of the gambling challenge. On the DEQ scale, High Recreational Gamblers reported increased drug-like “effect” and “high” compared to the Non-Gamblers. High Recreational Gamblers were also found to report increased drug-like “effects” compared to Non-Gamblers. There was also a visual trend indicating that High Recreational Gamblers reported increased “want more” in comparison to controls. Our study further show that High Recreational Gamblers revealed a tendency towards an increase of the stimulatory effects on the ARCI Benzedrine scale. Even though this result was in line with previous outcomes in the study, it was a modest result.

These results also have support in the literature on alcohol. High-risk groups show larger responses to alcohol than low-risk groups under many circumstances in both the acute stimulation and intoxication to an alcohol challenge (Newlin and Thomson 1990; Newlin et al. 2010; Brkic et al. 2016). Still, very few studies have studied the subjective effects of gambling in high and low risk groups. However, Leary and Dickerson (1985) found that provocation with a gambling or neutral stimuli prior to playing on a poker machine, led high frequent gamblers to show both increased subjective and autonomic arousal, and an even greater arousal was shown by the high frequency players. This study did not include a control group with non-players. Further, Moodie and Finnigan (2005) investigated arousal in frequent and infrequent fruit machine players, as well as non-gamblers. They found an increase in heart rate arousal in frequent players in comparison to controls.

Further, in the present study we did not find significant differences in any of the other measures taken (i.e. liking; want more) on the DEQ and the other stimulant measures on the ARCI or at the objective measure blood pressure. In the field of human psychopharmacology, both alcohol and amphetamine challenge studies shows that the neurochemical effects of the drugs are dose dependent and that the subjective effects are correlated to dose (Holdstock and de Wit 2001; Brkic et al. 2016). Studies with time dependent gambling challenges and preferred choice of gambling preference may have an impact in future experiments. Even though the results were non significant, we did observe trends of High Recreational Gamblers demonstrating increased liking and want more on the DEQ scale compared to the Non-Gamblers. Acute gambling challenge studies are still initially in the beginning phase as not many studies of this kind have been done. Yet, a number of findings suggest that an episode of gambling can induce stimulative drug-like effects that closely resemble a psychostimulant drug effect (Zack and Poulos 2004, 2009).

The cut of limits for High Recreational Gamblers versus Low Recreational Gamblers in our study was based on Currie et al. (2008) who validated the nature of low risk gambling using Canadian epidemiological gambling prevalence data by comparing the dose response relationship between risk of harm and level of gambling intensity. After a series of analysis exploring risk curves for gambling they found that a persons who gambles for more than 60 min at a time are at an increased risk for gambling related harm. Low-risk gambling limits appeared at a frequency of one time per week with an upper limit for dollars spent between $33 and $85 per month. The low risk individuals in our study gambled for about $75 a month and on average 17 min a week and the high-risk individuals for more than $300 a month almost 3.5 h/week. Yet, a large British Gambling Prevalence Survey found that gambling related harms such as dependence and social harms, were reported even among those who spend little time gambling (a mean of 30 min month) and spend less money (mean expenditure $15/month) than problem gamblers did (Canale et al. 2016). This study demonstrates that even a person that plays with little money and time can experience incentives and may therefore be at risk. All together, risk of harm from gambling appears to increase with greater gambling intensity regardless of gender, age or other demographic factors (Currie et al. 2008, 2012). Very few studies have collected such data as we did in the present study and divided a low-risk from a high-risk gambler by time spent gambling. Therefore, it is meaningful to further study high gamblers versus low gamblers based on time spend gambling also in future studies.

Although this study had several meaningful strengths, including a group of non-gambling individuals, comparable participant’s characteristics including gender and in first attempt unite a well know field of drug research to the field of gambling studies, several limitations should also be mentioned. First, the participants were recreational gamblers. One of the predictors of a vulnerability to a drug problem is the quality and magnitude to an individual’s response to a certain drug (Newlin and Thomson 1990). It is meaningful to map out early acute responses such as in this study in a non-clinical sample. However, future research is needed to assess the effects of a laboratory gambling challenge in a clinical sample. Second, our sample was small and quite homogenous. It is possible that the effect of the gambling challenge would have produced significant effects on several other stimulative variables on the DEQ and the ARCI if the sample size would not have been limited. This is supported by the fact that the trends towards increased subjective differences between recreational gambler vs non-gamblers was greater in the group of recreational gamblers. Even though by studying the second hypothesis with three groups, which made the sample size even smaller, the result stayed the same. Third, we did not control for the gambling challenge. It is therefore unclear if a control challenge would modify the interpretation of the effects of the slot machine in recreational gamblers. In alcohol research, heavy drinkers have repeatedly been found to show increased stimulatory effects compared to light drinkers (King et al. 2011). A non-blind control session where the participants for example would play solitaire, would not accurately blind or control for the subjective effects induced by the slot machine. We believed that in this preliminary study by only comparing gamblers with the non-gamblers we achieve a comparison for the rewarding properties for the behavioral gambling challenge. We aim to in future studies to include a control session. Fourth, in general, stimulatory effects of a drug is thought to be more rewarding than sedative effects and therefore increases the risk for future use and abuse (King et al. 2011, 2014) and a drugs ability to induce a striatal dopamine release is the mechanisms underlying stimulatory effects of a drug. In our study, we cannot state that High Recreational Gamblers are at increased risk for gambling addiction due to the stimulation induced by the gambling challenge. Our result should be interpreted with caution and we can only state that those who gamble for more than three hours a week show increased stimulatory effect of gambling compared to non-gamblers.

In sum, despite these limitations, the present study was preliminary in nature and its main purpose was to test the subjective effects of a gambling challenge in recreation gamblers. Our study demonstrated that high recreational gamblers show increased stimulatory effects to gambling, which indicates a uniquely predictive behavior, not identified in this group before.
